# Supplementary material for: Label-free real-time imaging of mitochondrial matrix volume changes and permeability transition in living cells
Source: bioRxiv. 2026 May 17:2026.05.15.725497. Preprint. [Version 1] doi: 10.64898/2026.05.15.725497 (PMC13192729; doi:10.64898/2026.05.15.725497)
Supplement: Supplement 1 [file NIHPP2026.05.15.725497v1-supplement-1.pdf]

bioRxiv preprint doi: https://doi.org/10.64898/2026.05.15.725497; this version posted May 17, 2026. The copyright holder for this preprint (which was not certified by peer review) is the author/funder, who has granted bioRxiv a license to display the preprint in perpetuity. It is made available under a CC-BY 4.0 International license.

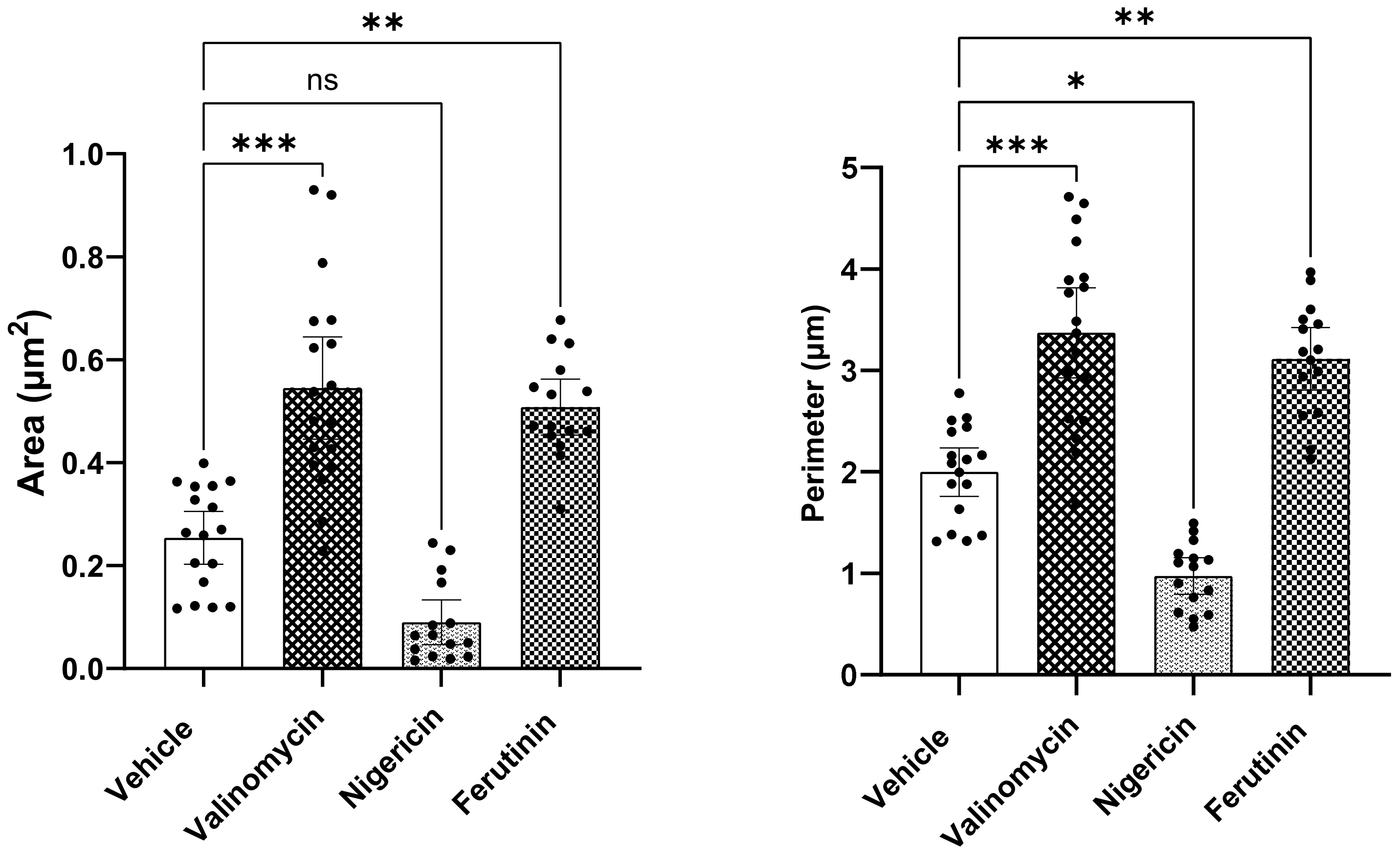

Supplementary Figure 1. Quantification of the EM images demonstrate increase in mitochondrial area (A) and perimeter (B) following valinomycin and ferutinin treatment indicating organelle swelling.

bioRxiv preprint doi: https://doi.org/10.64898/2026.05.15.725497; this version posted May 17, 2026. The copyright holder for this preprint (which was not certified by peer review) is the author/funder, who has granted bioRxiv a license to display the preprint in perpetuity. It is made available under a CC-BY 4.0 International license.

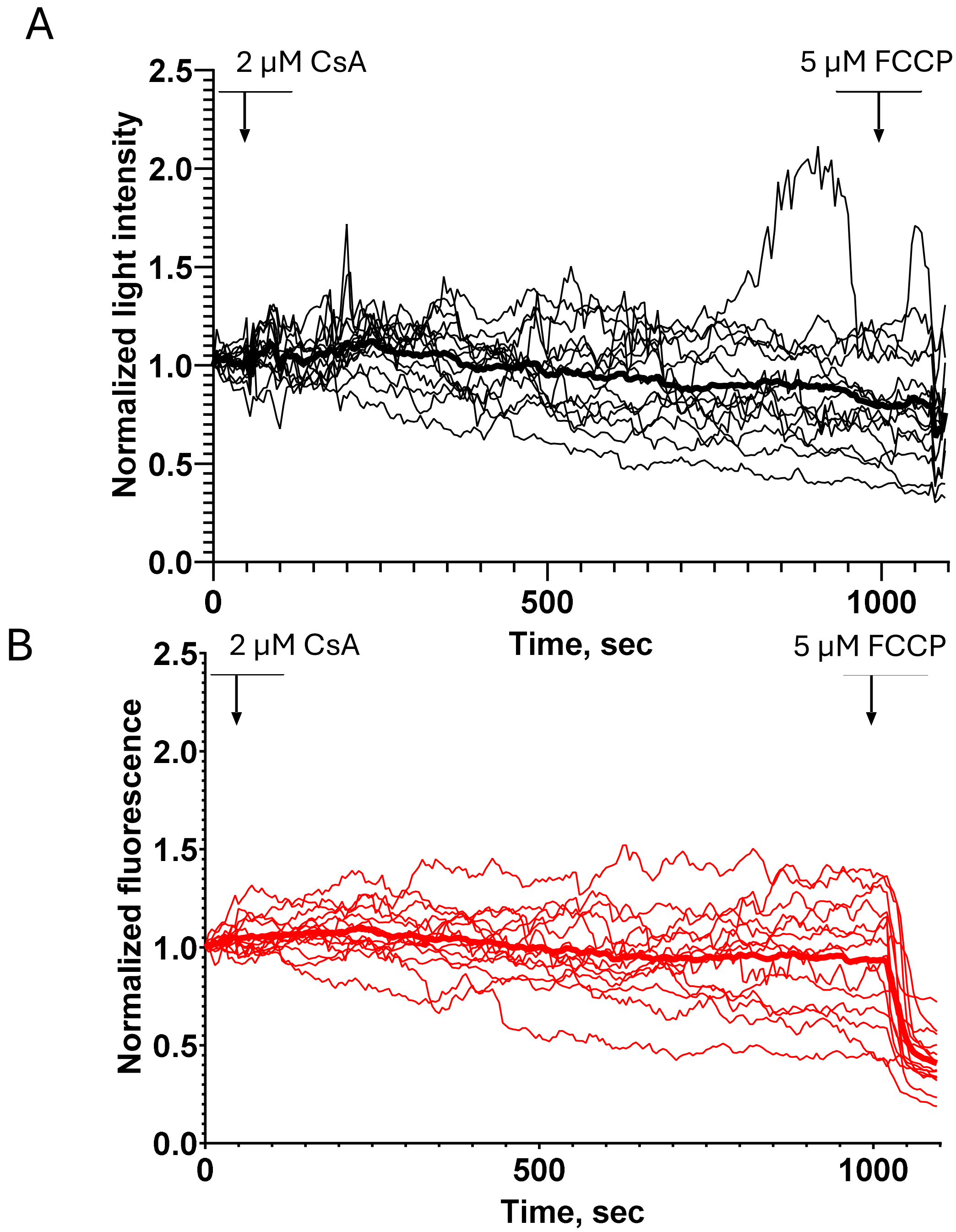

Supplementary Figure 2. Light scattering (A) and membrane potential (B) measurements of the HAP1 wild-type cells. Note lack of membrane depolarization.

bioRxiv preprint doi: https://doi.org/10.64898/2026.05.15.725497; this version posted May 17, 2026. The copyright holder for this preprint (which was not certified by peer review) is the author/funder, who has granted bioRxiv a license to display the preprint in perpetuity. It is made available under a CC-BY 4.0 International license.

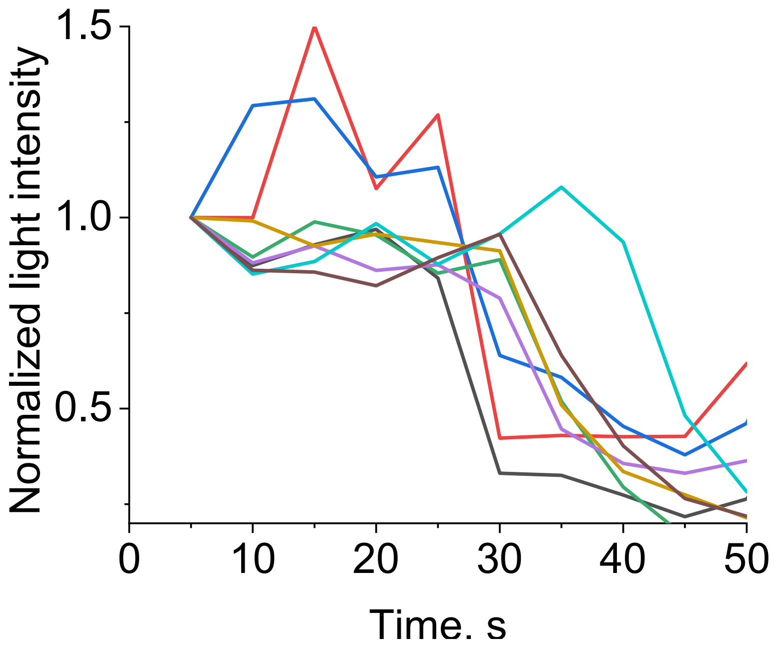

Supplementary Figure 3. Heterogeneous PTP dynamics in a single cell. Each trace originates from different ROI taken within the same cell. Note the onset of PTP occurs at different timepoints in some populations complete as early as at 30 sec (black trace) and as late as at 50 sec (magenta trace).
